# Unique Action of Interleukin-18 on T Cells and Other Immune Cells

**DOI:** 10.3389/fimmu.2018.00763

**Published:** 2018-04-20

**Authors:** Kenji Nakanishi

**Affiliations:** Department of Immunology, Hyogo College of Medicine, Hyogo, Japan

**Keywords:** interleukin-18, Th1, interferon-γ, interleukin-4, innate-type allergy, interleukin-33, ILC2

## Abstract

Interleukin (IL)-18 was originally discovered as a factor that enhances interferon (IFN)-γ production by anti-CD3-stimulated Th1 cells, particularly in association with IL-12. IL-12 is a cytokine that induces development of Th1 cells. IL-18 cannot induce Th1 cell development, but has the capacity to activate established Th1 cells to produce IFN-γ in the presence of IL-12. Thus, IL-18 is regarded as a proinflammatory cytokine that facilitates type 1 responses. However, in the absence of IL-12 but presence of IL-2, IL-18 stimulates natural killer cells, NKT cells, and even established Th1 cells to produce IL-3, IL-9, and IL-13. Thus, IL-18 also facilitates type 2 responses. This unique function of IL-18 contributes to infection-associated allergic diseases. Together with IL-3, IL-18 stimulates mast cells and basophils to produce IL-4, IL-13, and chemical mediators such as histamine. Thus, IL-18 also induces innate-type allergic inflammation. IL-18 belongs to the IL-1 family of cytokines, which share similar molecular structures, receptors structures, and signal transduction pathways. Nevertheless, IL-18 shows a unique function by binding to a specific receptor expressed on distinct types of cells. In this review article, I will focus on the unique features of IL-18 in lymphocytes, basophils, and mast cells, particularly in comparison with IL-33.

## Preface

I worked with Dr. William E. Paul from 1981 to 1984 in Laboratory of Immunology (LI), National Institutes of Allergy and Infectious Diseases, National Institutes of Health. As he was a laboratory chief at LI, I assumed he would have a large laboratory and research group. However, his laboratory was relatively small. Furthermore, he only had three postdoctral fellows (Anthony DeFranco, John Kung, and Maureen Howard) at my first visit. Nevertheless, he was regarded as a giant in the Immunology field. Indeed, he was a real giant, and also an outstanding mentor. Bill was a person who was glad to advise or supervise researchers when asked. Thus, young scientists with big dreams wanted to work with him. Weekly morning group meetings and one-to-one meetings with Bill were always exciting and helpful, and I learned a lot from him. Everybody respected him. He was a very kind and gentle boss. He was also an extremely intelligent man with striking creativity. But, perhaps most, I liked his shyness, because I am Japanese. I studied the functional roles of IL-4, IL-5, and IL-2 for growth and differentiation of B cells. After publishing one paper in *J Immunol* and two papers in *J Exp Med*, I left NIH and returned to Japan. I missed Bill and my friends at LI very much.

## Introduction

In Japan as a physician-scientist, I had several opportunities to learn that sepsis remains a common life-threatening disorder. Patients with high serum endotoxin levels did not necessarily develop lethal shock. Furthermore, patients with low serum endotoxin levels sometimes died of septic shock. Thus, we simultaneously measured the serum levels of endotoxin and interleukin (IL)-6, because lipopolysaccharide (LPS) induces IL-6 production *in vivo*. We found that there were at least two groups: an endotoxin shock susceptible group, characterized by high serum IL-6 level with low serum LPS level, and an endotoxin-resistant group, characrized by low serum IL-6 level with high serum LPS level. These findings indicated the presence of certain limiting factors that determined the sensitivity of patients to endotoxin shock. I learned that priming with heat-killed *Propionibacterium acnes* (*P*. *acnes*), a Gram-positive bacterium, or BCG increased the sensitivity of animals to the lethal effect of LPS. Thus, with Tomohiro Yoshimoto, my long-term collaborator, I studied the mechanism for how *P. acnes* increase the responsiveness of mice to LPS. We found that *P. acnes* priming rendered mice highly susceptible to the lethal effect of LPS by enhanced production of IL-1 and/or tumor necrosis factor-α (TNF-α) as well as increased responsiveness to the stimulation with IL-1 and/or TNFα.

After publishing these results (1) in 1992, I observed the very interesting phenomenon that *P. acnes*-primed BALB/c *nu/nu* mice were resistant to LPS-induced lethal shock, and instead most of them died of fulminant hepatitis through apoptosis-mediated hepatocytotoxicity. My colleagues, Haruki Okamura and Hiroko Tsutsui, demonstrated this severe liver injury was prevented by administration of a neutralizing anti-IL-18 antibody (2). These experiments were my first exposure to the unique action of IL-18, which forms the long-term target of my investigations and the main theme of this manuscript. In this review, I will initially describe animal models of LPS-induced diseases, and then describe the actions of IL-18 on T cells and other immune cells, as the major topic of the manuscript. Finally, I will compare the actions of IL-18 and IL-33 in various aspects. Pathological roles of IL-18 in various diseases, including hepatic, metabolic, inflammatory, allergic, and autoimmune diseases, are also documented in previous (3, 4) and recent (5, 6) reviews.

## Animal Models of LPS-Induced Diseases

### Susceptibility to LPS-Induced Endotoxin Shock

Mice primed with *P. acnes* markedly increased production of IL-1 and TNFα in response to LPS. Furthermore, these mice were highly susceptible to the lethal shock-inducing effect of IL-1 and/or TNFα (1). We tried to identify the limiting cells for LPS sensitivity. As *P. acnes*-primed BALB/c *nu/nu* mice were resistant to LPS-induced lethal shock, we examined the LPS susceptibility of these mice after reconstitution with splenic T cells from wild-type mice (7). We found that BALB/c *nu/nu* mice reconstituted with T cells became highly susceptible to LPS shock after *P. acnes* treatment and systemic administration of *P. acnes* induced development of Th1 cells in wild-type mice as well as in BALB/c *nu/nu* mice reconstituted with splenic T cells (7). Furthermore, IL-12p40-deficient mice or interferon (IFN)-γ-deficient mice were highly resistant to sequential treatment with *P. acnes* and LPS (7). Thus, IFN-γ-producing Th1 cells play an important role in determining host sensitivity to LPS shock (7).

### Susceptibility to LPS-Induced Liver Injury

The liver has a potent immune system (3). It contains residential immunocompetent cells with self-renewing ability, such as liver NK cells, extrathymically developed T cells, thymically developed CD4^+^NKT cells, expressing CD4 and NK cell markers, and a limited T-cell antigen receptor repertoire, and Kupffer cells, tissue macrophages. With my long-term colleague Kiyoshi Matsui, I demonstrated that hepatic CD4^+^NKT cells in non-treated wild-type mice promptly produced large amounts of IL-4 and IFN-γ upon stimulation with immobilized anti-CD3 *in vitro* (8). However, administration of heat-killed *P. acnes* in-duced hepatic CD4^+^NKT cells to increase IFN-γ production, but decrease IL-4 production upon anti-CD3 stimulation *in vitro* (8). These effects were attributable to the action of IL-12 from *P. acnes*-elicited Kupffer cells, suggesting a role for Kupffer cells in regulation of immune responses in the liver (3, 8). As noted above, most mice sequentially treated with *P. acnes* and LPS developed lethal shock, while the surviving mice suffered from liver injury. Meanwhile, BALB/c *nu/nu* mice sequentially treated with *P. acnes* and LPS developed severe liver injury. However, this severe liver injury was prevented by administration of a neutralizing anti-IL-18 antibody (2). Furthermore, *P. acnes*-primed IL-18-deficient mice did not develop liver injury upon LPS challenge (9, 10). However, we found that administration of IL-18-induced liver injury in *P. acnes*-primed IL-18-deficient mice by inducing Fas ligand expression and TNFα production in hepatic NK cells (3, 11). Based on these findings, we concluded that the development of thymic T cells into Th1 cells and hepatic CD4^+^NKT cells into predominant IFN-γ-producing cells was important for induction of LPS-driven endotoxin shock and LPS-induced liver injury in *P. acnes*-primed mice, respectively (7, 8).

## Overview of the IL-18/Interleukin 18 Receptor (IL-18R) System

Interleukin-18 was originally designated IFN-γ-inducing factor (IGIF), because it was first identified through its capacity to induce IFN-γ production by anti-CD3-stimulated Th1 cells (2, 12).Okamura and colleagues discovered this activity in sera or liver extracts from mice sequentially treated with *P. acnes* and LPS (2, 4). Based on the homology of its amino acid sequence to that of IL-1β, and its shared β-pleated sheet structure with IL-1β (2), IL-18 was classified into the IL-1 family of cytokines (13, 14). IL-18 is produced as a biologically inactive precursor, pro-IL-18, that is localized in the cytoplasm and requires proteolytic processing for secretion as active IL-18 (2–4). In collaboration with K. Kuida (Vertex, USA), S. Taniguchi (Shinsyu University, Japan), and J. Tschopp (University of Lausanne, Switzerland), we demonstrated that cleavage of pro-IL-1β and pro-IL-18 into mature IL-1β and IL-18, respectively, depended on the action of intracellular cysteine protease caspase-1, produced in the NLRP3 inflammasome consisting of pattern recognition receptor NLRP3 (NACHT-LRR and pyrin domain-containing protein 3), adaptor molecule ASC (apoptosis-associated speck-like protein containing a caspase recruitment domain), and pro-caspase-1 (15–18). However, we also found that Fas ligand treatment stimulated Fas-expressing Kupffer cells or macrophages to produce active IL-18 in a caspase-1-independent manner, indicating the presence of some other caspase-mediated pathways for IL-18 secretion (11). A recent study revealed that Fas mediated noncanonical IL-1β and IL-18 maturation *via* caspase-8 (19). In addition, IL-18 can be activated in an inflammasome-independent manner by proteases, such as proteinase 3 (20), chymase (21), and granzyme B (22).

Interleukin 18 receptor is composed of the inducible IL-18Rα chain (IL-1R-related protein or IL-1R5) and constitutively expressed IL-18Rβ chain (IL-1R-associated protein-like or IL-R7) (4, 6). The IL-18Rα and IL-18Rβ chains are members of the IL-1R family, and their cytoplasmic domains contain a TLR/IL-1R (TIR) domain, a common domain shared by toll-like receptors (4–6, 13, 14). IL-18Rα is an IL-18-binding receptor that, upon stimulation with IL-18, forms an IL-18 high-affinity binding heterodimer with IL-18Rβ that mediates intracellular signal transduction (23, 24). The cytoplasmic TIR domains of the IL-18R complex interact with myeloid differentiation factor 88 (MyD88), a signal adaptor containing a TIR domain, *vi*a a TIR–TIR interaction (4–6, 13, 14, 25). In collaboration with S. Akira (Osaka University), we revealed that the major biological activities of IL-18 were completely abrogated in MyD88-deficient mice (25). In turn, MyD88-induced events resulted in successive activation of nuclear factor-κB and mitogen-activated protein kinase by association with the signal adaptors IL-1R-associated kinase (IRAK) 1, IRAK4, and TNF receptor-activated factor 6, respectively (25), eventually leading to expression of appropriate genes, such as *Il4, Il13, Ifnγ, Tnf*, and *Fasl*, involved in cell differentiation, growth, survival, and apoptosis (2–6, 8–14, 23–26).

Interleukin-18-dependent cell activation can be inhibited at least by two distinct molecules. One is the naturally occurring IL-18-binding protein (IL-18BP) (27). Because IL-18BP binds to IL-18 with high affinity (400 pM), it can downregulate IL-18-induced cell responses, such as IL-18-induced Th1 cell IFN-γ production. Another inhibitor is the anti-inflammatory cytokine IL-37, a member of the IL-1 family of cytokines (28). Although IL-37 binds to IL-18Rα with low affinity, the resulting complex inhibits recruitment of IL-18Rβ, thereby abolishing signal transduction *via* IL-18R. Furthermore, this complex induces recruitment of IL-1R8, an orphan receptor of the IL-1 family formerly known as SIGIRR, to form a tripartite complex (IL-37/IL-18Rα/IL-1R8), which does not bind MyD88, but instead induces anti-inflammatory signal into the cell. Thus, IL-18 activity is inhibited by these two distinct inhibitors (6).

## Mechanism for LPS-Induced Liver Injury in *P. acnes*-Primed Mice

**Figure 1:**
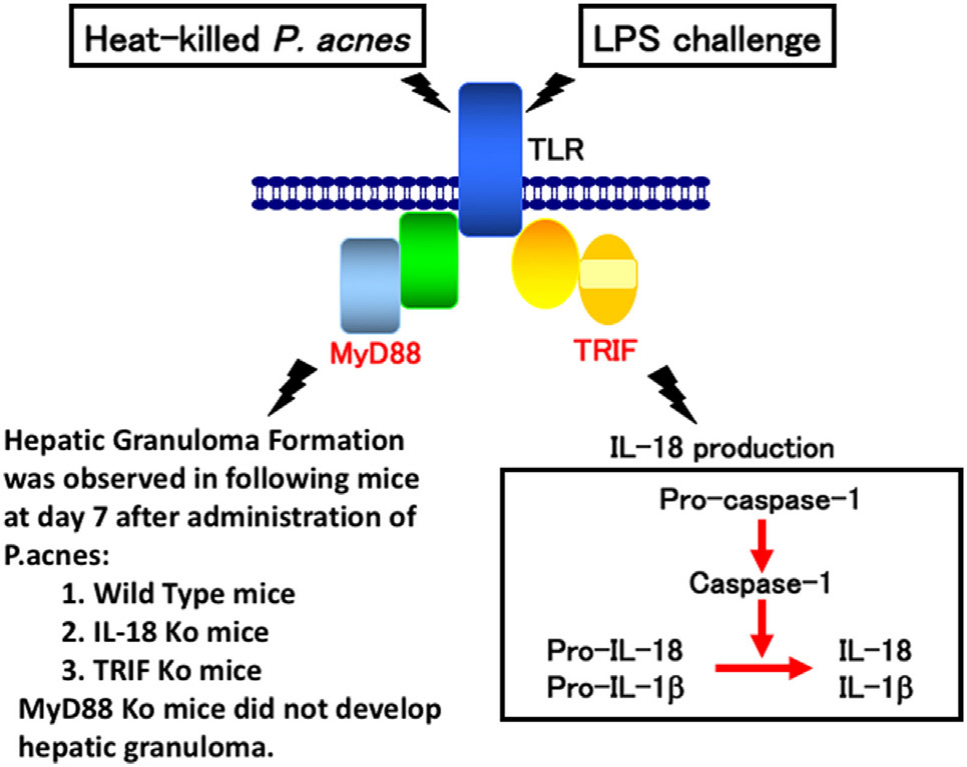
A proposal model for lipopolysaccharide (LPS)-induced liver injury in *Propionibacterium acnes* (*P. acnes*)-primed and LPS-challenged mice. Wild-type mice or mice deficient for interleukin (IL)-18, MyD88, or TRIF were administered with heat-killed *P. acnes* and examined for their hepatic granuloma formation at day 7 after this treatment. Only *P. acnes*-primed MyD88 did not develop hepatic granuloma at day 7 after treatment, suggesting that *P. acnes* treatment induces hepatic granuloma in a MyD88-dependent manner, but TRIF-independent manner. Although TRIF-deficient mice normally developed hepatic granulomas after *P. acnes* treatment, they could not release IL-18 or develop liver injury, suggesting that LPS TRIF-dependently activated caspase-1 *via* NLRP3 inflammasome. And, resultant IL-18 induces liver injury by induction of interferon-γ, FasL, and tumor necrosis factor-α.

Consistent with a previous report (29), wild-type mice primed with *P. acnes* developed dense granulomas in the liver. These mice also developed acute liver injury and elevated serum IL-18 level after challenge with a sublethal dose of LPS (2–5). Although *P. acnes*-primed IL-18-deficient mice exhibited dense granulomas, similar to the liver of *P. acnes*-primed wild-type mice, they did not develop liver injury after LPS treatment (10, 11). In contrast, MyD88-deficient mice primed with *P. acnes* showed very poor hepatic granuloma formation and produced an undetectable level of IL-18 upon LPS challenge (17). This failure to produce IL-18 in response to LPS was not caused by a loss of potential of MyD88-deficient Kupffer cells to produce IL-18,because MyD88-deficient Kupffer cells were able to secrete IL-18 in response to LPS *in vitro* (30). Thus, *P. acnes* treatment induced hepatic granuloma formation in a MyD88-dependent manner and LPS stimulated Kupffer cells to produce IL-18 in a MyD88-independent manner (Figure 1). Next, we examined the contribution of TRIF (TIR domain-containing adapter inducing IFN-β) to *P. acnes*-induced hepatic granuloma formation and LPS-induced IL-18 secretion. In contrast to MyD88-deficient mice, *P. acnes*-primed TRIF-deficient mice showed normal development of hepatic dense granuloma, but did not release IL-18 and, therefore, did not develop liver injury (17). Thus, we concluded that *P. acnes* treatment induced hepatic granuloma formation in a MyD88-dependent manner and that subsequent LPS challenge induced caspase-1 activation in a TRIF-dependent manner in the NLRP3 inflammasome and induced IL-18 release, eventually leading to liver injury (17) (Figure 1).

## Several Topics for the Unique Functions of IL-18

### IFN-γ Production

**Figure 2:**
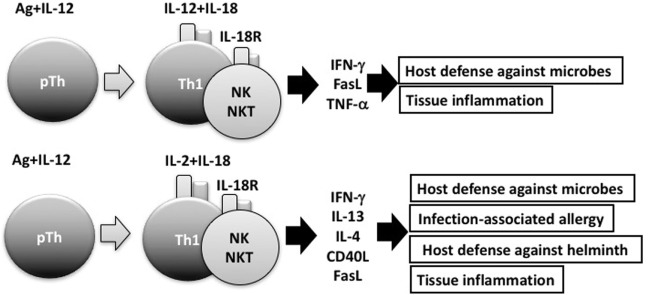
Interleukin (IL)-18 facilitates both Th1 response and Th2 response. In combination with IL-12, IL-18 stimulates Th1 cells, natural killer (NK) cells, and NKT cells to produce robust interferon (IFN)-γ, resulting in the clearance of intracellular microbes. However, without IL-12, but with IL-2, IL-18 induces Th1 cells, NK cells, and NKT cells to produce IL-13 and IFN-γ both of which are involved in host defense response or in infection-associated allergy. NKT cells stimulated with IL-2 and IL-18 express CD40L and produce IL-4, thereby inducing B cells to produce IgE.

Consistent with its original discovery as an IFN-γ-inducing factor, IL-18 can induce IFN-γ production by natural killer (NK) cells and Th1 cells that express IL-18R (2, 4) (Figure 2). However, IL-18 also synergizes with IL-12 to induce marked IFN-γ production by various cell types, including nonpolarized T cells, NKT cells, dendritic cells, macrophages, and B cells, through reciprocal induction of expression of their corresponding receptors (4). It is well known that B cells produce IgG1 and IgE when stimulated with anti-CD40 and IL-4. To our surprise, a combination of IL-12 and IL-18 inhibited IL-4-dependent IgG1 and IgE production, but enhanced IgG2a production by inducing IFN-γ production in B cells stimulated with IL-12 and IL-18 (31). Indeed, IL-12-stimulated B cells expressed IL-18R and strongly produced IFN-γ in response to IL-18, particularly in association with IL-12 (23). We also found that naïve Th cells stimulated with antigen (Ag) and IL-12 or IL-4 developed into IL-18R-expressing Th1 or ST2-expressing Th2 cells, respectively (23, 24, 32). Thus, expression of IL-18R and ST2 can be a convenient cell marker for Th1 and Th2 cells, respectively.

### Th2 Cytokine Production by Mast Cells and Basophils Stimulated With IL-18

**Figure 3:**
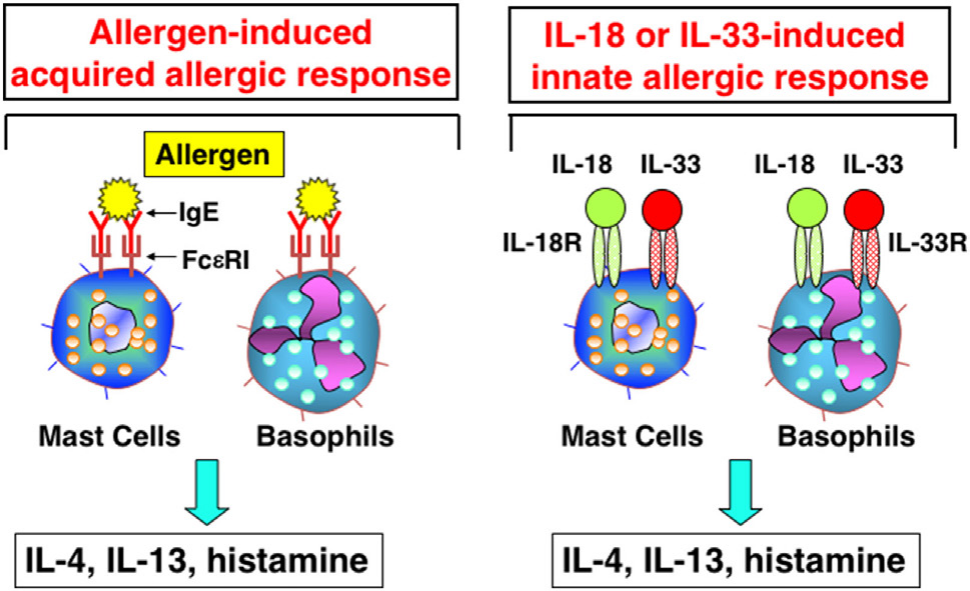
Interleukin (IL)-18 and IL-33 induce innate allergic response by stimulation of mast cells and basophils. Like mast cells or basophils stimulated with Ag/IgE complex, mast cells and basophils, expressing IL-18R and IL-33R, produce IL-4, IL-13 and chemical mediators, after being stimulated with IL-18 and IL-33, respectively.

In 1989, Marshall Plaut and Bill Paul reported in *Nature* that, upon cross-linkage of FcεR1 with Ag/IgE complex, mast cells, and basophils produce Th2 cytokines, including IL-4 and IL-13 (33). Thus, I was interested to know whether mast cells and basophils also had the potential to produce IFN-γ after stimulation with IL-12 and IL-18. I discussed this matter with Bill, and he said “I am very interested in what will happen.” Thus, Tomohiro and I started collaboration with Bill. We found that basophils and mast cells derived by culture of bone marrow cells with IL-3 for 10 days expressed the IL-18Rα chain and produced large amounts of IL-4 and IL-13 in response to stimulation with IL-3 and IL-18 (34). These were unexpected results, but turned out to be very important findings. To our disappointment, however, mast cells and basophils never produced IFN-γ in response to various combinations of IL-3, IL-18, and IL-12 (34). As the combination of IL-18 and IL-3 stimulated basophils and mast cells to produce histamine and Th2 cytokines, we speculated that IL-18 could induce allergic inflammation without assistance from the Ag/IgE complex. Thus, we reported a new aspect of IL-18 as an inducer of Th2 cytokine production from basophils and mast cells in 1999 (34) (Figure 3). Later, I became interested in the capacity of basophils to produce IL-4 upon cross-linkage of FcεR1 with Ag/IgE complex. Surprisingly, we detected expression of MHC class II molecules on basophils (35). Thus, we examined the capacity of basophils pulsed with Ag/IgE complex to induce development of naïve Th cells into Th2 cells. We found that basophils had the capacity to induce development of Th2 cells (35). Although we were still unable to determine the physiological role of basophils as APCs, we believe that further studies will demonstrate such an activity in basophils.

### Innate-Type Allergic Inflammation

After publication of the paper on Th2 cytokine production by basophils and mast cells stimulated with IL-3 and IL-18, I speculated that IL-18 may have the potential to induce IL-4 production by CD4^+^ T cells and/or CD4^+^NKT cells. I found that injection of a mixture of IL-12 and IL-18 increased serum IgE levels in helminth-infected IFN-γ-deficient mice. Most surprisingly, daily administration of IL-18 in particular with IL-2 induced a marked increase in serum IgE levels in a CD4^+^ T cell- and IL-4/IL-4R/STAT6-dependent manner (36). Furthermore, CD4^+^NKT cells stimulated with IL-2 and IL-18 increased their CD40 ligand expression and IL-4 production. In addition, these activated CD4^+^NKT cells induced development of B cells into IgG1- and IgE-producing cells. Consistent with these findings, transgenic mice overexpressing human caspase-1 in keratinocytes, established by Hitoshi Mizutani (Mie University), produced IL-18 and IgE in their sera, and also spontaneously developed atopic dermatitis (AD)-like skin lesions (37). Disruption of STAT6, required for IL-4 signal transduction, abolished IgE production without affecting the skin manifestations. In contrast, disruption of IL-18 in caspase-1 transgenic mice diminished their chronic dermatitis almost completely, although they still produced significant amounts of IgE. Thus, overproduction of IL-18 by keratinocytes induced AD-like skin lesions even in the absence of IgE and IgG1 (37). Based on these results, we designated this IL-18-induced allergic inflammation an innate-type allergic inflammation.

In the presence of IL-2, but absence of IL-12, IL-18 stimulated NK cells, CD4^+^ NKT cells, and splenic CD4^+^ T cells to produce IL-3, IL-9, and IL-13 (26, 36) (Figure 2). Because IL-3 and IL-9 induce mucosal mastocytosis, we examined whether the animals developed mucosal mastocytosis after treatment with IL-2 and IL-18. We found that C57BL/6 mice pretreated with IL-18 and IL-2 developed mucosal mastocytosis with high levels of serum mMCP1, an activation marker of MMC, and became able to promptly expel the intestinal nematode *Strongyloides venezuelensis*. Thus, IL-18 is important for expulsion of intestinal nematodes by induction of mucosal mastocytosis, and we published these results in *J Exp Med* (38).

## Th1 Cells Produce IFN-γ and IL-13 in Response to Ag and IL-18

It is well established that IL-18 increases IFN-γ production by anti-CD3-stimulated Th1 cells, particularly in association with IL-12 (2, 4). Furthermore, endogenous IL-18 is required for host defense against intracellular microbes, such as *Listeria monocytogenes, Cryptococcus neoformans*, and *Leishmania major*, because IL-18-induced IFNγ activated the infected macrophages sufficiently to kill these pathogens (4, 39, 40). However, we had not examined the possibility that IL-18-stimulated Th1 cells can produce Th2 cytokines. Thus, we stimulated established ovalbumin (OVA)-specific Th1 cells with OVA and/or IL-18 and found that OVA plus IL-18-stimulated Th1 cells produce both a Th1 (IFN-γ) and Th2 cytokines (IL-9, IL-13) (41) and additional IL-2 stimulation enhanced production of Th2 cytokines (Figure 2).

Next, we examined whether IL-18 acts on memory Th1 cells to induce airway inflammation and airway hyperresponsiveness (AHR) in naïve host mice. In 2002, Nobuki Hayashi and Bill Paul developed a method to establish both resting Th1 and Th2 memory cells (42). Nobuki performed a wonderful study after coming back to my laboratory from the LI. To avoid a background response of host-derived T cells, he administered newly polarized OVA-specific Th1 or Th2 cells into naïve mice and allowed them to adopt a resting memory phenotypy *in vivo*. Intranasal administration of OVA induced airway inflammation and AHR only in mice that received Th2 cells (41). However, mice that received Th1 cells developed airway inflammation and AHR after intranasal administration of both OVA and IL-18 (41). Th1 cells stimulated with OVA and IL-18 became harmful cells, which we designated “super Th1 cells,” that produced IFN-γ and IL-13, the combination of which induced difficult bronchial asthma (41). Nobuki further demonstrated that naïve mice having resting Th1 memory cells developed severe bronchial asthma in response to nasal administration of OVA plus LPS instead of IL-18. He also revealed that endogenous IL-18 from LPS-stimulated bronchial epithelial cells was responsible for inducing severe bronchial asthma. He published these results in 2007 (43). This prominent feature of IL-18 can explain the mechanism for infection-associated allergic diseases (44) (Figure 2).

Intriguingly, after several rounds of stimulation with Ag, IL-2 plus IL-18, Ag-specific Th1 cells were found to differentiate from cells producing both IL-13 and IFN-γ into cells producing IL-13, but little IFNγ. My colleague Masakiyo Nakahira verified that GATA3 was essential for induction of IL-13 in Th1 cells after stimulation of these cells with Ag, IL-2, and IL-18 (45). Thus, IL-18 has the potential to induce plasticity of established Th1 cells (41, 43–45) (Figure 2).

## Similarities and Differences Between IL-18 and IL-33

Interleukin-33, a member of the IL-1 cytokine family, is a ligand of ST2. IL-33 is synthesized as a full-length active form, stored in the nucleus, and released from cells when they receive mechanical damage or become necrotic (46–48). IL-18 is an immunoregulatory cytokine (4) that acts with IL-12 to stimulate Ag-stimulated Th1 cells to produce IFN-γ (2, 4, 12), but acts with IL-2 to stimulate the same cells to produce both a Th1 (IFN-γ) and a Th2 cytokine (IL-13) (41, 43–45) (Figure 2). In contrast, IL-33 has the capacity to induce Ag-stimulated Th2 cells to increase production of Th2 cytokines (IL-4, IL-5, and IL-13) (46–48), suggesting that IL-33 plays an important role in induction of allergic responses.

**Figure 4:**
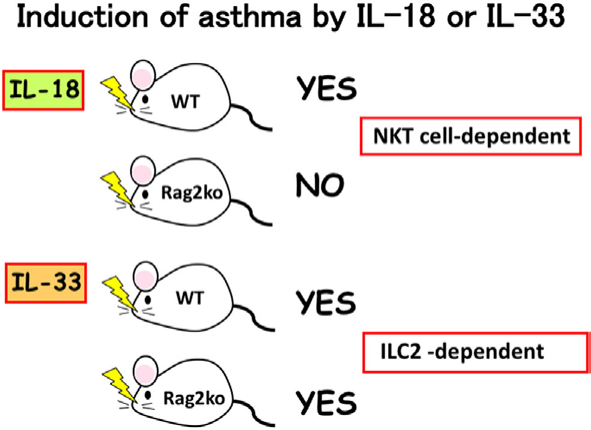
Induction of bronchial asthma by intranasal administration of interleukin (IL)-18 or IL-33. As natural killer (NK)T cells constitutively express IL-18R, intranasal administration of IL-18 into wild-type mice, but not into Rag2Ko mice induced bronchial asthma by induction of IL-4 and IL-13 from NKT cells. In contrast, intranasal administration of IL-33 into wild-type mice and Rag2Ko mice equally induce bronchial asthma, because Rag2Ko mice are equipped with ILC2 which express IL-33R and produce IL-13 in response to IL-33.

We found that mast cells and basophils express both IL-18R and IL-33R and produce IL-4 and IL-13, when stimulated with IL-3 plus IL-18 or with IL-33, respectively (34, 49) (Figure 3). Therefore, IL-18 and IL-33 have very similar effects on mast cells and basophils. Moreover, IL-18 and IL-33 show similar pathological effects on the lungs. Nasal administration of IL-2 and IL-18 induced AHR, pulmonary eosinophilia, and goblet cell hyperplasia in wild-type mice, but not in Rag2-deficient mice (50) (Figure 4). However, nasal administration of IL-33 induced the same changes in both wild-type mice and Rag2-deficient mice (49) (Figure 4). Thus, IL-2 plus IL-18 induced these pulmonary changes in a NKT cell-dependent manner, while IL-33 treatment induced the same changes in a NKT cell-independent and innate cell-dependent manner (Figure 4). Moro et al. (51) and Neill et al. (52) showed that natural helper cells (NH cells) or nuocytes, currently designated group 2 innate cells (ILC2s), express IL-33R, and produce IL-5 and IL-13 in response to IL-33.

My long-term colleague Koubun Yasuda revealed the mechanism for how IL-33 induced the above pulmonary changes in the absence of acquired immunity. He showed that IL-33 treatment increased the number of ILC2s and that the IL-33-activated-ILC2s induced pulmonary eosinophilia and goblet cell hyperplasia by producing IL-5 and IL-13 in a T-cell-independent manner (53). Thus, IL-33 plays an important role in the induction of ILC2-dependent allergic diseases. Furthermore, he found that infection with the intestinal nematode *S. venezuelensis*, which transiently migrates into the lungs, increased the number of IL-33-producing alveolar epithelial type II cells in the lungs of wild-type mice and Rag2-deficient mice (53). Thus, both types of mice infected with *S. venezuelensis* developed eosinophilic inflammation and goblet cell hyperplasia in their lungs (Loeffler syndrome) (53). Therefore, IL-33 production and release in the lungs is very important for induction of pulmonary eosinophilic inflammation during nematode infection (53–55).

## Author Contributions

The author confirms being the sole contributor of this work and approved it for publication.

## Conflict of Interest Statement

The author declares that the research was conducted in the absence of any commercial or financial relationships that could be construed as a potential conflict of interest.
